# A Novel G16B09-Like Effector From *Heterodera avenae* Suppresses Plant Defenses and Promotes Parasitism

**DOI:** 10.3389/fpls.2019.00066

**Published:** 2019-02-08

**Authors:** Shanshan Yang, Yiran Dai, Yongpan Chen, Jun Yang, Dan Yang, Qian Liu, Heng Jian

**Affiliations:** Department of Plant Pathology and MOA Key Laboratory of Pest Monitoring and Green Management, China Agricultural University, Beijing, China

**Keywords:** *Heterodera avenae*, effector, G16B09 family, suppressed plant defense, PTI, ETI

## Abstract

Plant parasitic nematodes secrete effectors into host plant tissues to facilitate parasitism. In this study, we identified a G16B09-like effector protein family from the transcriptome of *Heterodera avenae*, and then verified that most of the members could suppress programmed cell death triggered by BAX in *Nicotiana benthamiana*. Ha18764, the most homologous to G16B09, was further characterized for its function. Our experimental evidence suggested that *Ha18764* was specifically expressed in the dorsal gland and was dramatically upregulated in the J4 stage of nematode development. A *Magnaporthe oryzae* secretion system in barley showed that the signal peptide of Ha18764 had secretion activity to deliver mCherry into plant cells. *Arabidopsis thaliana* overexpressing *Ha18764* or *Hs18764* was more susceptible to *Heterodera schachtii*. In contrast, BSMV-based host-induced gene silencing (HIGS) targeting *Ha18764* attenuated *H. avenae* parasitism and its reproduction in wheat plants. Transient expression of *Ha18764* suppressed PsojNIP, Avr3a/R3a, RBP-1/Gpa2, and MAPK kinases (MKK1 and NPK1^Nt^)-related cell death in *Nicotiana benthamiana*. Co-expression assays indicated that Ha18764 also suppressed cell death triggered by four *H. avenae* putative cell-death-inducing effectors. Moreover, Ha18764 was also shown strong PTI suppression such as reducing the expression of plant defense-related genes, the burst of reactive oxygen species, and the deposition of cell wall callose. Together, our results indicate that Ha18764 promotes parasitism, probably by suppressing plant PTI and ETI signaling in the parasitic stages of *H. avenae*.

## Introduction

*Heterodera avenae* is an important cereal cyst nematode (CCN) that infects wheat, barley, and oat crops in the cereal-growing regions worldwide. The wheat yield losses caused by *H. avenae* can range widely, from 10% up to 100% in some infected fields (Bonfil et al., 2004; Smiley et al., 2017). The infective second-stage juvenile (J2) of this cyst nematode penetrates the root tips of its host plant and migrates intracellularly toward the vascular cylinder, then it inserts its stylet into a cell and induces the infusion of surrounding cells, resulting in the formation of a multinucleate syncytium (Jones, 1981). Because cyst nematodes are obligate sedentary endoparasites that feed from the syncytia until their reproduction is complete, they closely interact with their host plants. The same with other plant pathogens, nematodes can secrete effector proteins to regulate host plant cellular processes to promote their parasitism. Most of these effectors are produced in the esophageal glands and are delivered into plant cells via the nematode’s hollow stylet.

Plants have developed the immune system to protect them from pathogen attacks. A comprehensive overview of the multifaceted co-evolutionary plant–pathogen interactions is conveyed in the “zigzag” model (Jones and Dangl, 2006). In it, plants respond to pathogen infection by using a two-branched immune system. The first branch recognizes microbe/pathogen-associated molecular patterns (MAMPs/PAMPs) to trigger MAMP/PAMP-triggered immunity (MTI/PTI) responses, such as callose deposition, the burst of reactive oxygen species (ROS), and the induction of defense-related gene expression (Luna et al., 2011; Mendoza, 2011). To enhance their survival, infecting pathogens deliver effectors that interfere with PTI for successful parasitism. The second plant immune branch recognizes one effector by a resistance protein and activates an effector-triggered immunity (ETI) response, usually resulting in hypersensitive cell death at the infection site (Cui et al., 2015). Pathogen isolates might gain new effectors to suppress ETI, and this relationship illustrates the dynamic co-evolution between plants and their pathogens (Tsuda and Katagiri, 2010).

Nematode effectors play a wide variety of roles in root penetration, suppression of host defenses, and the formation and maintenance of feeding sites (Haegeman et al., 2012). In recent years, those effectors capable of suppressing plant immunity have garnered increasing attention (Chronis et al., 2013; Jaouannet et al., 2013; Diaz-Granados et al., 2016). Recently, a number of effectors were found to be capable of suppressing plant defense responses in sedentary endoparasitic nematodes (Favery et al., 2016). These include the root-knot nematode-secreted effectors Mi-CRT, MiMSP40, MiSGCR1, MiISE6, Mj-TTL5, Mh265, MeTCTP, MgGPP, and MgMO237, as well the cyst nematode-secreted effectors Hs10A06, GrSPRYSEC19, GrCEP12, GrVAP1, Ha-ANNEXIN, and HgGLAND18 (Hewezi et al., 2010; Postma et al., 2012; Chronis et al., 2013; Jaouannet et al., 2013; Lozano-Torres et al., 2014; Chen et al., 2015, 2017; Lin et al., 2016; Niu et al., 2016; Noon et al., 2016; Gleason et al., 2017; Zhuo et al., 2017; Chen J. et al., 2018; Nguyen et al., 2018; Shi et al., 2018). Moreover, the increasing availability of genome sequences for plant parasitic nematodes now promotes to identify more and more effectors (Abad et al., 2008; Opperman et al., 2008; Kikuchi et al., 2011; Thorpe et al., 2014; Eves-van Den Akker et al., 2016). In particular, many effectors are actually related proteins encoded by gene families (Cotton et al., 2014). One notable family is that of the SPRY domain gene (approximately 300 sequences) in *Globodera pallida*, which has several secreted protein members that function as selective suppressors of defense-related cell death in plants (Mei et al., 2015; Diaz-Granados et al., 2016). The HYP effectors comprise a large gene family with continual expression and they play an important role in plant–nematode interactions (Eves-van Den Akker et al., 2014). The diversity in the effector family may be due to selection pressures to evade recognition by the host.

The effectors G16B09, 4D06, and related proteins (here referred to as the “G16B09 family”) were first identified in a gland-cell cDNA library of *H. glycines*, which had been built by micro-aspirating the cytoplasm from esophageal gland cells of parasitic nematode stages (Gao et al., 2003). Since then, two new G16B09-family members were likewise identified from *H. glycines* (Noon et al., 2015). Therefore, 11 distinct member proteins of the G16B09 family are currently known in the nematode *H. glycines*. In *G. pallida*, the G16B09 family is considered among the largest of its gene families, for which 39 members have been identified so far (Thorpe et al., 2014). The mRNAs of all these members are expressed specifically within the dorsal gland cell of parasitic stages of *H. glycines* or *G. pallida*, indicating their likely contribution to syncytium induction and formation. Nevertheless, all these members are also novel transcripts with no homology to any reported genes in public databases, rendering them the “pioneers” designation. Nor were functional domains detected in any of them using computational tools. Characterizing the functions of this complex effector family would provide crucial information for better understanding nematode–plant interactions.

In this study, we identified a G16B09 family from *H. avenae*. Then we characterized one G16B09-like effector protein (here named “Ha18764” after its transcriptome identification number) with a significant virulence function in nematode–plant interactions. We determined that Ha18764 provides this nematode with a significant virulence function. Furthermore, we present several lines of ancillary evidence showing this novel effector most likely works by suppressing PTI and/or ETI responses in host plants, to facilitate *H. avenae* parasitism. This study provided an experimental clue for further investigating the functions of G16B09-like effector proteins.

## Materials and Methods

### Nematodes and Plants

*H. avenae* was propagated on wheat (*Triticum aestivum* cv. Aikang 58) using second-stage juveniles (J2s) hatched from cysts, previously collected from a wheat field in Qingdao, China. The pre-parasitic (pre-J2s) were collected by hatching the cysts at 15°C after at least 4 weeks incubation at 4°C. To obtain the parasitic nematodes, infected wheat roots were obtained at 5, 20, and 30 days post inoculation (dpi), cut into sections, and digested at 28°C by shaking at 160 rpm in a 6%-cellulose water solution overnight (Chen et al., 2015). The parasitic-stage juveniles (par-J2) were obtained directly from 5 dpi. The third-stage (J3) and fourth-stage (J4) juveniles were, respectively, obtained from 20 dpi and 30 dpi. Females were collected by hand picking them from the wheat root surfaces. In our laboratory, *H. schachtii* nematodes were propagated on the beet *Beta vulgaris* L. heir pre-J2s were collected by hatching the cysts at 25°C.

Wheat and barley (*Hordeum vulgare* cv. E9) were grown in a greenhouse at 22°C under a 16-h light/8-h dark cycle. *Nicotiana benthamiana* was grown in a growth chamber at 25°C under a 14-h light/10-h dark cycle. The *Arabidopsis thaliana* plants were grown on solidified Murashige and Skoog (MS) medium with 2% sucrose under sterile conditions, or grown in potting soil in a growth chamber at 23°C (16-h light/8-h dark cycle).

### Gene Amplification and Sequence Analysis

Genomic DNA and total RNA were prepared from freshly hatched pre-J2s using, respectively, the TIANamp Micro DNA Kit and RNAprep Pure Micro Kit (Tiangen, Beijing, China). The cDNA was synthesized from total RNA by using the SMART^®^ MMLV Reverse Transcriptase (Takara, Tokyo, Japan) according to the manufacturer’s instructions. Based on our *H. avenae* transcriptome data (Yang et al., 2017), the DNA sequence of *Ha18764* and the cDNA of all the genes were cloned by PCR amplification using their specific primers (Supplementary Table S1). To search for homologies, the *Ha18764* cDNA sequence was BLASTed against the GenBank database or the published genomic database of potato cyst nematodes (Cotton et al., 2014; Eves-van Den Akker et al., 2016). The *H. schachtii Ha18764*-like sequence was obtained by PCR using the primers *HgG16B09*cds-F/R and cDNA template. All the primers used in this study are listed in Supplementary Table S1.

To identify the effector gene homologs, a local, command line BLAST was carried out against the *H. avenae* transcriptome sequence, using an E-value threshold of 10^-5^ and with the low complexity filtering turned off (Thorpe et al., 2014). The sequence homology of the predicted proteins was then analyzed using DNAMAN, Clustal X v2.0, and BoxShade software tools. We searched for the conserved domain and secretory signal peptide (SP) with NCBI CD-Search^1^ and SignalP v4.1^2^, respectively. Prediction of putative transmembrane domains were obtained according to TMHMM^3^. Finally, the *in planta* subcellular localization was predicted using PSORT^4^.

### Developmental Expression Analysis and *in situ* Hybridization

Total RNA was extracted and cDNA synthesized from different stages nematodes as described above. Using the primer pairs *Ha18764*qPCR-F/*Ha18764*-qPCR-R and *HaGAPDH*-1-F/*HaGAPDH*-1-R, respectively, qRT-PCR amplified the *Ha18764* gene and the endogenous reference gene *HaGAPDH*-1, with the reagent SYBR Premix Ex Taq II (Tli RNaseH Plus; Takara, Tokyo, Japan) on a ABI PRISM 7500 system (Applied Biosystems, United States). Triplicate PCR reactions for each cDNA sample were carried out, and the assay itself consisted of three technical replicates. The obtained data were analyzed following the 2^-ΔΔCt^ method.

For the *in situ* hybridization, *H. avenae* J2s were hatched in leachates of wheat root and collected. The primers *in-situ-Ha18764*-F/*in-situ-Ha18764*-R (Supplementary Table S1) were used to synthesize the DIG-labeled antisense and sense (negative control) cDNA probes (Roche, United States) by an asymmetric PCR. Hybridization was conducted as described previously (de Boer et al., 1998), and examined under a BX51 microscope (Olympus, Japan). Three independent experiments were performed.

### Subcellular Localization in *N. benthamiana*

The *Ha18764* gene without its SP-encoding region was amplified, by using the primer pairs *Ha18764*dsp-F/*Ha18764*dsp-R that, respectively, contained *Sal* I and *Xma* I restriction enzyme sites (Supplementary Table S1). The ensuing amplified fragments were cloned into the corresponding sites in the p35SeGFP vector to express the eGFP fusion protein. The empty vector served as the control. The construct was confirmed by sequencing, after which it was transformed into *Agrobacterium tumefaciens* strain EHA105. The recombinant *A. tumefaciens* carrying p35SeGFP-Ha18764 or p35SeGFP was infiltrated into *N. benthamiana* leaves, as described by Chen et al. (2015). After 48 h, infiltrated leaves were visualized under laser confocal fluorescence microscope (Nikon Eclipse TE300, Tokyo, Japan) at an excitation wavelength of 488 nm. Three independent experiments were performed.

### Validation of the Predicted Signal Peptide in Barley

To assess whether the SP of Ha18764 is secretory, a live-cell imaging approach with slight modifications (Park et al., 2012) was developed to localize Ha18764 in barley. First, the *Ha18764* gene with its SP-encoding region was amplified using the primers *Ha18764*VaF/R containing the *Hind* III and *Bam* HI restriction enzyme sites (Supplementary Table S1), respectively. These amplified fragments were cloned into the respective sites in the pRP27-mcherryNLS vector to express the mCherry fusion protein. The empty vector was used as a negative control. The ensuing constructs were transformed into the protoplast of the fungal *Magnaporthe oryzae* strain p131. This recombinant p131 carrying the constructs was cultured in an OTA medium (oatmeal tomato agar medium) at 26°C for 10 days (Yang et al., 2010). Next, the spores were suspended in 5% Tween 20, to an appropriate concentration of 10–15 spores/100 μL, then inoculated to *in vitro* leaves of 10-day-old barley. After inoculation, the barley leaves were incubated at 26°C for about 27 h under wet and dark conditions. Nuclei were stained with DAPI (4′,6-diamidino-2-phenylindole; Katsuhara and Kawasaki, 1996) and visualized under a BX61 microscope (Olympus, Japan). Three independent experiments were performed.

### Silencing of *Ha18764* by BSMV-HIGS and the *H. avenae* Infection Assay

The specificity of selected gene fragments of *Ha18764* was confirmed by a BLAST search with NCBI data and our *H. avenae* transcriptome data. The specific *Ha18764*RNAi fragment was amplified by PCR using the primer pairs *Ha18764*RNAi-F/*Ha18764*RNAi-R (Supplementary Table S1). Barley stripe mosaic virus-medicated host-induced gene silencing (BSMV-HIGS) and nematode infection assay were conducted as previously described (Yuan et al., 2011; Chen et al., 2015). For the infection assay, approximately 300 J2s of *H. avenae* were inoculated to wheat plants (*n* = 16), then the number of nematodes in the roots were counted at 7 dpi, and females at 50 dpi were also counted. Meanwhile, the expression level of *Ha18764* in nematodes from wheat inoculated by BSMV:Ha18764 relative to that of the blank negative control (BSMV:00) and the negative control (BSMV:eGFP) were determined by qPCR. This experiment was independently repeated three times. Independent-samples *t*-tests or one-way ANOVA (Duncan’s test for pairwise means), conducted in SPSS v13.0, were used to analyze the differences between the treatment groups.

### Suppression of Immune-Associated Cell Death

This assay were conducted as previously described (Chen C. et al., 2018). Coding sequences (without SPs) of each gene of *H. avenae* were constructed into the PVX vector pGR107 (Jones et al., 1999), fused with the 3× flag-tag at the N-terminus by an In-Fusion HD Cloning Kit (Clontech, United States). Using the same kit, the necrosis elicitor gene *psojNIP* (Qutob et al., 2002) was constructed into the pGR107 vector with an HA-tag fused at the C-terminus. Four candidate effector genes of *H. avenae*—namely *isotig16511*, *isotig16978*, *isotig19390*, and *isotig12969*—capable of triggering cell death in *N. benthamiana* leaves were constructed into the vector pND108 with a HA tag. These constructs were confirmed by sequencing and then transformed into the *A. tumefaciens* strain GV3101 for infiltration. The pGR107:GFP with a flag-tag, and both pGR107:NbMKK1 and pGR107:NbNPK1^Nt^ with an HA tag had been generated in our prior work (Chen et al., 2015). Other researchers kindly provided us with the construct pGR107-Bax, the vectors expressing Avr3a, R3a, Gpa2, or Rbp-1, as well as the empty vector PMD1 (see “Acknowledgments” section).

Suppression of cell death as mediated by different elicitors in *N. benthamiana* leaves was assayed as already described elsewhere (Wang et al., 2011). The *A. tumefaciens* cells carrying *Ha18764*, or other family genes, were infiltrated into the leaves of *N. benthamiana*. After 24 h, *A. tumefaciens* cells carrying the elicitor genes were infiltrated into the same site, while the *A. tumefaciens* strain carrying the *Ha18764* or *GFP* gene and a buffer was infiltrated alone as the controls. These assays were independently repeated at least three times, with three to six *N. benthamiana* plant replicates inoculated each time (to three leaves per plant). Photographs of the infiltrated leaves of *N. benthamiana* were obtained ca. 5 days since the last infiltration was made or after decolorizing their leaves (by boiling in alcohol for 20 min). To verify gene expression, a Western blotting protocol was followed as described previously (Chen et al., 2015).

### Generation of Transgenic *Arabidopsis thaliana* Plants and the *Heterodera schachtii* Infection Assay

The *Ha18764*- or *Hs18764*-coding cDNA sequence (without SP) was amplified by the primer pairs 1300-Ha18764-F/1300-Ha18764-R or 1300-Hs18764-F/1300-Hs18764-R (Supplementary Table S1) and these generated sequences were inserted into the *Hind* III *or Kpn* I respective sites of the vector pSuper1300, respectively. Then the ensuring constructs were transformed into *A. tumefaciens* GV3101, which was used to transform the *A. thaliana* ecotype Col-0 by the floral dip method. Seeds of the transformants were collected and stored at 4°C for 7 days, then selected by Hygromycin B in an MS solidified medium containing 2% sucrose for ca. 14 days, then transplanted into soil. Homozygous T3 seeds collected from the T2 lines were used.

For the infection assay, 14-day-old *A. thaliana* plants (*col-0, col-0* containing *Ha18764* or *Hs18764*) were inoculated with 300 pre-J2s of *H. schachtii*. For each host plant, their respective number of infected nematodes in the roots was counted at 14 dpi, (*n* = 20, respectively). This experiment was independently repeated three times. Independent-samples *t*-tests or one-way ANOVA (Duncan’s test) were used to analyze the differences in infection between the treatment groups by SPSS software.

### Defense-Related Gene Expression in Transgenic *Arabidopsis*

To determine the expression levels of defense-related genes, 14-day-old *A. thaliana* seedlings were soaked in sterile water containing 10 μM of flg22. Total RNA were isolated from 50 mg of *Arabidopsis* seedlings after 4 h using the TRIzol RNA extraction reagent (Invitrogen, United States). Transcript abundances of *WRKY70*, *WRKY29*, *PR-1*, and *CYP81F2* were determined by RT-qPCR. *Arabidopsis actin* served as an internal control to normalize the gene expression levels. Each sample reaction was run in triplicate. Independent-samples *t*-tests or one-way ANOVA (Duncan’s test) were used to analyze the differences in transcript abundances.

### ROS Generation Analysis

Detection of the flg22-mediated oxidative burst was performed using a luminol-HRP-based chemiluminescence assay. In this assay, the *Ha18764*-coding cDNA sequence (without SP) was amplified by primer pairs 1132-Ha18764-F/1132-Ha18764-R (Supplementary Table S1), then subcloned into the *Bam*H I*/Sal* I restriction sites of the vector pYBA1132. The ensuing construct pYBA1132:Ha18764 was introduced into *A. tumefaciens* GV3101 (freeze-thaw method). Then the GV3101 containing either pYBA1132 or pYBA1132:Ha18764 was infiltrated into *N. benthamiana* leaves (4- to 5-week-old plants). At 36-h post-infiltration, leaf discs (4 mm diam.) were collected and incubated overnight in 100 μL of H_2_O, in a 96-sample microplate, and substituted by 100 μL elicitor master mix (100 μM luminol, 20 μg/ml horseradish peroxidase, 100 nM flg22). The plate was immediately put into the microplate reader, with ROS production monitored for 40 min (Sang and Macho, 2017). This assays were performed three times, with triplicate reaction for each sample.

### Callose Staining

This assay were conducted as previously described (Tran et al., 2017). Following treatment with 1 μM of flg22, 8-day-old *Arabidopsis* seedlings were cultivated on the ½-MS basal agar medium for 72 h. Their roots were fixed overnight in a solution containing 95% ethanol and acetic acid (3:1), followed by their rehydration in 70% ethanol for 1 h, 50% ethanol for 1 h, and distilled water for 1 h, and then treatment with 10% NaOH for 1.5 h at 37°C to make their root tissues transparent. Finally, the roots were incubated in a staining solution (0.01 % aniline blue, 150 mM K_2_HPO_4_, pH 9.5) for at least 1 h; root tips ca. 1–2 cm length were excised and mounted onto slides for callose observation under a Leica TCS SP8 microscope with UV light (excitation, 390 nm; emission, 460 nm). Images were photographed in the field of approximately 2 mm^2^ that captures the root area containing the root elongation zone. Callose deposits in 12 roots per treatment from three independent experiments were counted using ImageJ software^5^.

## Results

### Most G16B09 Family Effectors From *H. avenae* Suppress BAX-Triggered Programmed Cell Death (BT-PCD) in *N. benthamiana*

A BLAST search of our transcriptome data of *H. avenae* (Yang et al., 2017) examined 12 homologs of this nemtaode’s G16B09 effector family (Supplementary Figures S1, S7), using an E-value threshold of 10^-5^ (Thorpe et al., 2014). No domains, motifs, or features could be predicted from the sequences, which are identical to those in *H. glycines* and *G. pallida* (Cotton et al., 2014). Eight of the 12 homologs were successfully cloned—their GenBank accession numbers are listed in Supplementary Figure S1—and investigated for their role in suppressing plant defenses. Infiltration with the *Agrobacterium* carrying *Bax* alone triggered a typical PCD reaction, whereas infiltration with *GFP* did not suppress BT-PCD (Supplementary Figure S2A). Of the eight *H. avenae* candidate effector genes evaluated, seven could suppress BT-PCD (Supplementary Figures S2B–H), while one gene had negligible effects on suppressing BT-PCD or triggering cell death (Supplementary Figure S2I). Western blotting confirmed the expression of *H. avenae* candidate effector protein, eGFP, and BAX. This finding suggested that the G16B09-like family of *H. avenae* contribute to suppressing the host plant immune system.

**FIGURE 1:**
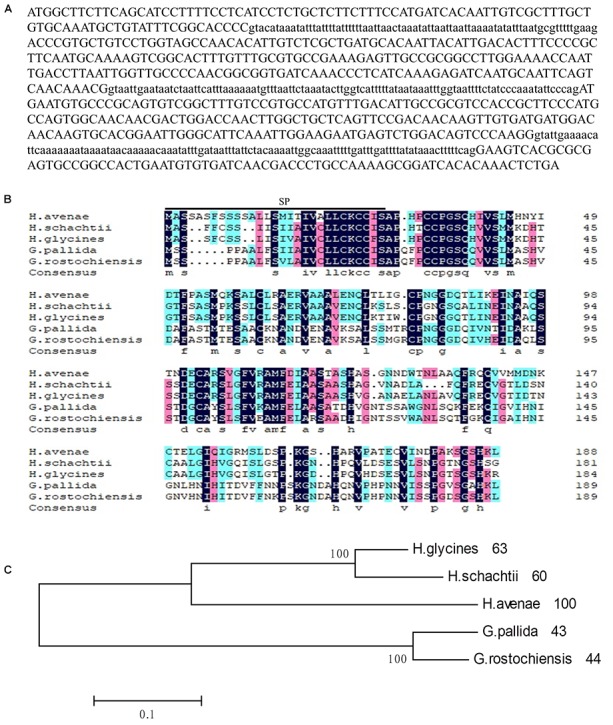
Sequence analysis of Ha18764 from the nematode *Heterodera avenae*. **(A)** The DNA sequence of *Ha18764*. Three introns are shown in lower-case letters. **(B)** Multiple sequence alignment of Ha18764 with homologs from other plant-parasitic nematodes. **(C)** Phylogenetic tree for Ha18764 and its homologs from some other cyst nematodes.

To further characterize the role of the G16B09 family from *H. avenae* in parasitism and plant defense suppression, the *isotig18764* (named *Ha18764* after its transcriptome identification number) with greatest amino acid similarity (61.9%) to G16B09 of *H. glycines* was selected for detailed study. An 844-bp genomic fragment of *Ha18764* was obtained, consisting of an open reading frame (ORF) of 567 bp separated by three introns of 75 bp, 98 bp, and 104 bp (Figure 1A), each having conserved 5′-GT-AG-3′ splice sites. *Ha18764* encodes 188 amino acids with a SP of 29 amino acids at its N-terminus (as predicted by the SignalP 4.1 server). This protein has no putative transmembrane domain (based on TMHMM Server v2.0) with a predicted molecular size of 19.94 kDa. According to the NCBI CD-search, Ha18764 has no conserved domain, motifs, or features. No nuclear localization signals were predicted for Ha18764 (according to the PSORT analysis).

For the alignment analysis, the homologs with highest similarity to *HgG16B09* from *G. pallida* and *G. rostochiensis* were obtained by a BLAST search against the public genome database (Cotton et al., 2014; Eves-van Den Akker et al., 2016). The homologous sequence from *H. schachtii*, here designated as *Hs18764* (GenBank Accession MH794364), was generated by PCR using the primers *HgG16B09cds*F/R (Supplementary Table S1). An alignment of deduced amino acid sequences of G16B09-like proteins from different nematode species is presented in Figure 1B. A consensus phylogeny tree based on the analyzed protein sequences divided them into three clades. Ha18764 showed 43–63% shared amino acid identity with other nematode homologs (Figure 1C).

### Ha18764 Is Expressed in the Dorsal Gland and Is Dramatically Up-Regulated in par-J4 of *H. avenae*

**FIGURE 2:**
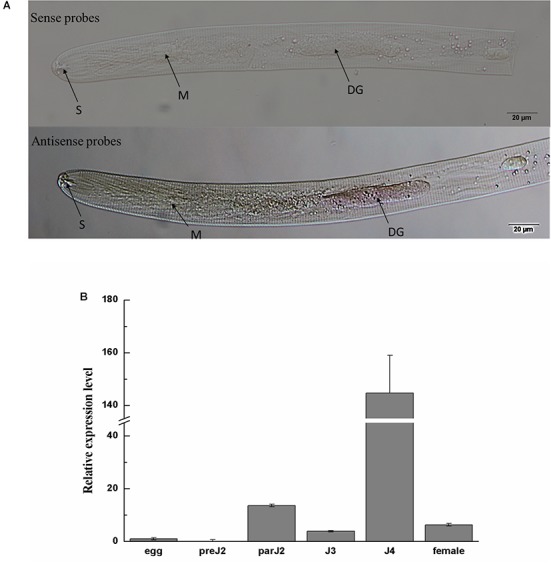
Spatial and developmental expression of *Ha-18764*. **(A)** Localization of *Ha18764* mRNA in the dorsal gland of pre-parasitic second stage juveniles of *Heterodera avenae* by *in situ* hybridization. The dorsal gland (DG), metacorpus (M), and stylet (S) are indicated with arrows. **(B)** Developmental expression pattern of *Ha18764*. The relative expression level of *Ha18764* was quantified using qPCR for six different *H. avenae* stages. The fold-change values were calculated using the 2^-ΔΔCt^ method and presented as the change in mRNA level in various nematode developmental stages relative to that of egg. Each column represents the mean (±SD) of three samples. This experiment was independently repeated three times, with consistent results. pre-J2, pre-parasitic second-stage juvenile; par-J2, J3, J4, parasitic second-, third- and fourth-stage juveniles, respectively.

*In situ* hybridization was performed to investigate the tissue localization of *Ha18764* in *H. avenae*. No signals were detected in *H. avenae* pre-J2s. However, when the pre-J2s were pre-treated with the leachates of wheat roots, signals were observed in the dorsal gland cells after the hybridization with the DIG-labeled antisense probe (Figure 2A). In the negative control (sense probe), no signals were detected in pre-J2s.

The expression level of *Ha18764* was determined by qPCR analysis for six developmental stages: egg, pre-J2, par-J2, J3, J4, and adult female. The expression level of *Ha18764* transcripts at the egg stage was set at a value of one, to serve as the baseline for examining the relative fold changes in later stages. *Ha18764* transcripts increased dramatically in the parasitic stages, reaching a maximum in the J4 stage that represented a 144-fold increase in expression (Figure 2B). These findings suggest Ha18764 may be secreted from dorsal gland cells and that it participates in parasitic stages, particularly in the later ones of *H. avenae* parasitism.

### Functional Validation of the Predicted Signal Peptide (SP) of Ha18764

**FIGURE 3:**
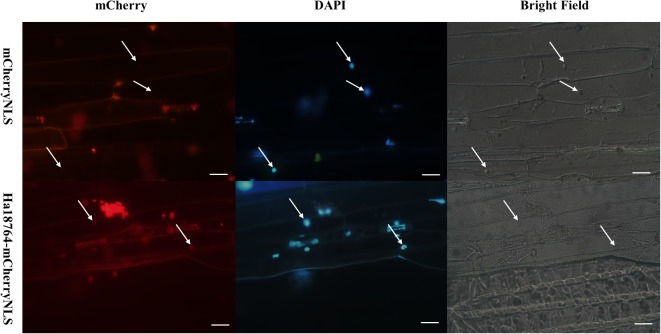
Functional validation of the signal peptide of Ha18764 using a *Magnaporthe oryzae* mCherry secretion assay. The SP of Ha18764 can guide secretion of the mCherry lacking a signal sequence into the plant; hence, red fluorescence was observable in the barley cell nucleus. *M. oryzae* strain p131 containing the mCherryNLS construct served as a negative control. Arrows indicate barley plant nuclei. Scale bar = 20 μm.

We have employed the yeast secretion system to verify the activity of the predicted SP of *H. avenae* effectors (Chen C. et al., 2018). However, the SP of Ha18764 was found to lack secretion activity in yeast cells (data not shown). Considering that secretion activity may differ between yeast and pathogens, a *Magnaporthe oryzae* secretion system was exploited to experimentally verify the secretion of Ha18764 (Park et al., 2012; Zhang et al., 2018). The *Ha18764* ORF (including the SP encoding sequence) was fused in-frame with the *mCherry* gene and a nuclear localization signal (NLS). The fusion constructs were transformed into *M. oryzae* strain p131, which was then used to inoculate the *in vitro* leaves of barley. In this assay, a functional SP could guide secretion of the mCherry fusion protein into barley cells during the infection of *M. oryzae*, with the fusion protein imported into the cell nucleus, targeted by the NLS; this should facilitate visualization of the translocated fluorescent protein by concentrating the fusion protein in the barley nucleus. Our results revealed that red fluorescence was observed only in the nucleus of barley inoculated with *M. oryzae* carrying the Ha18764-mCherry-NLS fusion construct (Figure 3). By contrast, no red fluorescence could be found in the barley nucleus inoculated with *M. oryzae* carrying the empty vector. The result indicated the secretion signal of Ha18764 guided mCherry to secrete into barley cells.

### Ha18764 Is Localized in the Whole Plant Cell

**FIGURE 4:**
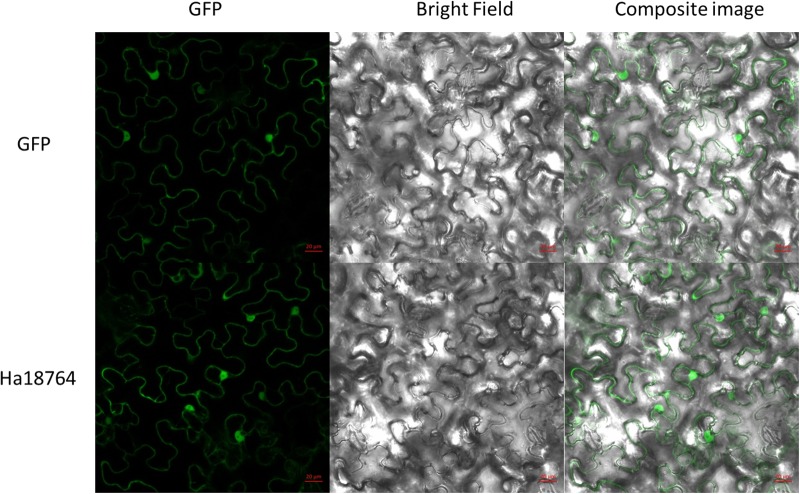
Subcellular localization of Ha18764 in *Nicotiana benthamiana* leaves. *Agrobacterium tumefaciens* cells carrying the Ha18764:GFP fusion contrast were infiltrated into *N. benthamiana* leaves with pCamv35S: GFP used as the control. Scale bar = 20 μm.

To investigate subcellular localization of Ha18764 in plant cells, a transient expression assay was performed with *N. benthamiana* leaves. The *Ha18764* coding sequence without its SP was fused in-frame with the enhanced green fluorescent protein (eGFP) and transformed into *Agrobacterium* for infiltration into *N. benthamiana* leaves. The transient expression of the fusion protein and eGFP alone showed the same accumulation of the GFP signal in both cytoplasm and nuclear (Figure 4). The nuclear accumulation of eGFP and Ha18764-eGFP may be due to its small size and passive diffusion of the fusion protein.

### Expression of *Ha18764* in *Arabidopsis* Improved Susceptibility to *H. schachtii*

**FIGURE 5:**
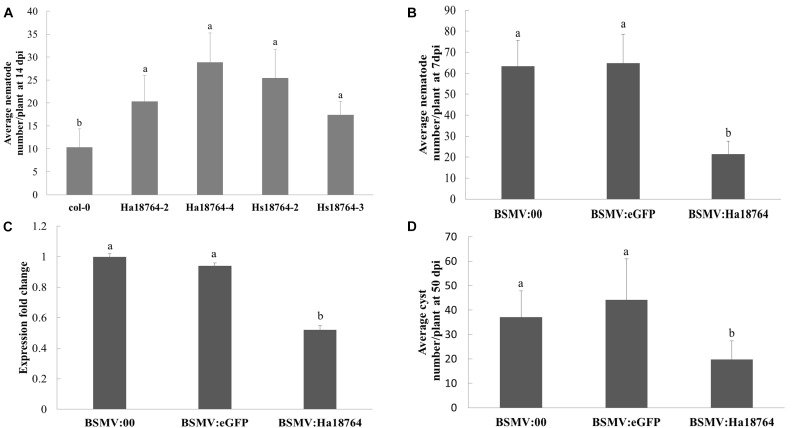
Effects of overexpression and host-derived RNAi of *Ha18764* on plant susceptibility to nematodes. **(A)** *Arabidopsis Ha18764* or *Hs18764* expression homozygous T3 lines increased the number of infected *H. schachtii* in roots compared to wild-type (WT) lines. **(B)** Relative expression level of *Ha18764* in *H. avenae* individuals collected on wheat inoculated by BSMV:18764 and BSMV:00 at 7 dpi (days post-infection). **(C)** Number of juveniles/plant in wheat roots at 7 dpi. **(D)** Number of females/plant at 50 dpi in wheat roots. Shown are means ± SD from *n* = 5–10 plants. The independent experiments were repeated thrice, with consistent results. Columns for the same time point or treatment marked with different letters are significantly different (*P* < 0.05) from each other.

Overexpressing an effector protein in the host plant is typically employed to investigate its involvement in parasitism. However, the efficacy of genetically transforming wheat is low and this process is slow, and *H. avenae* has a narrow host range. Therefore, to aid protein functional characterization, the model plant *Arabidopsis* was used because it is a host for *H. schachtii*, a close relative of *H. avenae*. Specifically, *Hs18764* was cloned and used as a homolog control; it shared a 71.4% sequence identity with *Ha18764*. Two independent homozygous T3 lines expressing either *Ha18764* or *Hs18764* transcripts were generated. Then *H. schachtii* was inoculated to determine the susceptibility of these transgenic *Arabidopsis* lines to nematode infection. The results showed that, at 14 dpi, either *Ha18764* or *Hs18764* transgenic lines were significantly more susceptible to *H. schachtii* infection than the wild-type *Arabidopsis* (*P* < 0.05), displaying average increases in numbers of nematodes per root that ranged from 96.0 to 178.1% and 68.0% to 145.1%, respectively, over the wild-type (Figure 5A). These results indicated that *Ha18764* plays an important role in nematode parasitism.

### BSMV-HIGS of *Ha18764* Impairs Nematode Parasitism

Barley stripe mosaic virus (BSMV) vectors are efficient vehicles for virus-induced gene silencing (VIGS) in wheat (Yuan et al., 2011). A novel approach, called “host-induced gene silencing” (HIGS), can silence a pathogen’s genes with BSMV-VIGS to interfere with its effective infection of wheat (Nowara et al., 2010; Yin et al., 2011). Recently, this system was successfully employed to silence nematode gene during parasitism of wheat (Chen et al., 2015). Here, the expression of *Ha18764* in nematodes recovered from wheat inoculated by BSMV:*Ha18764* showed a significant reduction compared with that from the controls BSMV:00 and BSMV:*eGFP* (*P* < 0.05) (Figure 5B). Accordingly, *H. avenae* infection of wheat inoculated by BSMV:*Ha18764* showed significant resistant to nematodes, displaying a 66.2% or 67.0% reduction in juveniles abundance per plant at 7 dpi (Figure 5C), and a 46.7% or 55.2% reduction in female abundance per plant at 50 dpi (Figure 5D), when compared, respectively, to the negative control BSMV:00 or to BSMV:*eGFP* (*P* < 0.05). These results provided further evidence for the important involvement of Ha18764 in nematode parasitism.

### Ha18764 Suppresses Immune-Associated Cell Death in *N. benthamiana*

**FIGURE 6:**
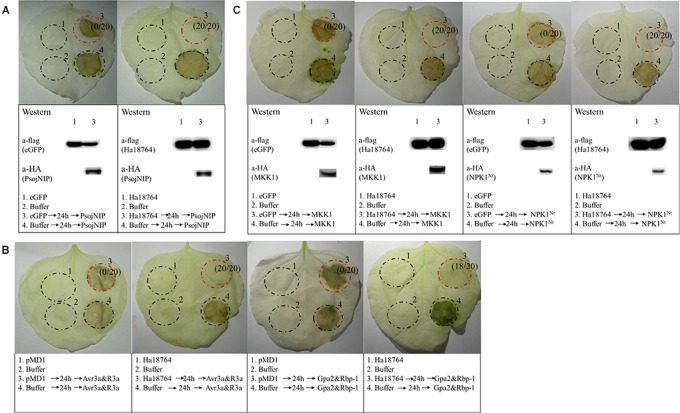
Ha18764 suppresses immune-associated cell death. **(A)** Ha18764 suppressed PsojNIP-triggered cell death. **(B)** Ha18764 suppressed the cell death triggered by either Avr3a/R3a or Gpa2/RBP-1. The empty pMD1 vector served as a negative control. **(C)** Ha18764 suppressed MAPK cascade-associated cell death. *Nicotiana benthamiana* leaves were infiltrated with buffer or *Agrobacterium tumefaciens* cells carrying Ha18764 or the negative control, either alone or 24 h prior to infiltration with *A. tumefaciens* cells carrying the elicitors. Photographs of infiltrated leaves were taken ca. 4 days after the last infiltration. Four circles marked with Roman numerals represented regions injected with different *A. tumefaciens*, which is indicated under the photographs. The numbers in parentheses indicate the proportion of infiltrated sites showing cell-death-suppressing symptoms. The original Western blotting images for the verification of gene expression were provided in Supplementary Figures S3, S4.

To further explore the possible role of Ha1874 in plant defense suppression, we tested for the suppression of cell death triggered by various elicitors. PsojNIP (Qutob et al., 2002), Avr3a/R3a (Abramovitch et al., 2003), RBP-1/Gpa2 (Sacco et al., 2009), and MAPK cascade-associated protein kinases (MKK1 and NPK1^Nt^) (Jin et al., 2002; Gao et al., 2008) were all used to investigate the ability of Ha18764 to influence cell death suppression during parasitism. At least three repeated experiments showed that Ha18764 suppressed cell death induced by any one of PsojNIP (Figure 6A), Avr3a/R3a or RBP-1/Gpa2 (Figure 6B), or MKK1 and NPK1^Nt^ (Figure 6C). This suggested Ha18764 is a potent suppressor of plant immune-associated cell death.

### Ha18764 Suppresses Cell Death Induced by Other *H. avenae* Putative Effectors in *N. benthamiana*

**FIGURE 7:**
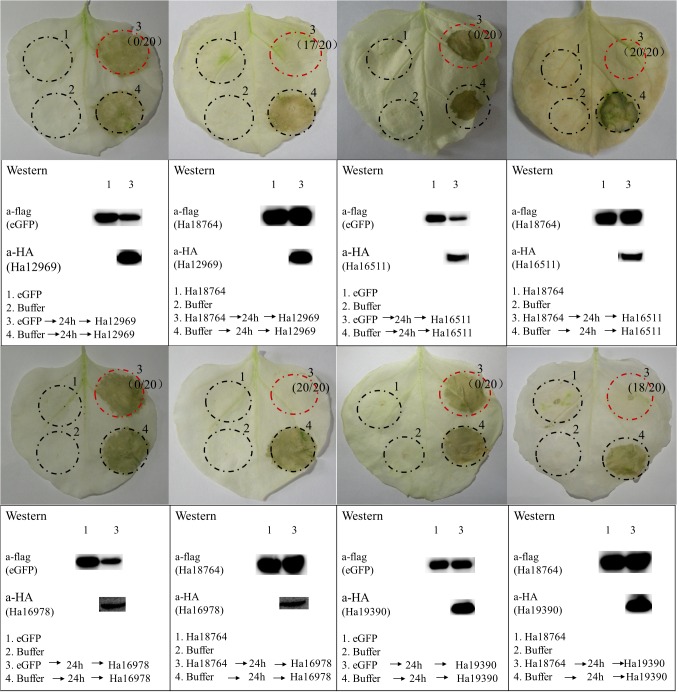
Ha18764 suppress cell death triggered by other candidate *Heterodera avenae* effectors in *Nicotiana benthamiana*. *Nicotiana benthamiana* leaves were infiltrated with buffer or *Agrobacterium tumefaciens* cells carrying Ha18764 or the negative control, either alone or 24 h prior to infiltration with *A. tumefaciens* cells carrying the elicitors. Photographs of infiltrated leaves were taken ca. 4 days after the last infiltration. Four circles marked with Roman numerals represented regions injected with different *A. tumefaciens*, which is indicated under the photographs. The numbers in parentheses are the proportion of infiltrated sites showing cell-death-suppressing symptoms. Results for the verification of gene expression by Western blotting are shown below. The original Western blotting images for the verification of gene expression were provided in Supplementary Figures S5, S6.

In the screening of the transient expression assay for *N. benthamiana* leaves, several *H. avenae* putative effectors could themselves trigger cell death (Chen C. et al., 2018). Hence, it was pertinent to test whether Ha18764 might also suppress cell death triggered by these cell-death-inducing effector candidates. Thus, four cell-death-inducing genes, *isotig16511*, *isotig16978*, *isotig19390* and *isotig12969*, were selected as cell death inducers and used for agroinfiltration tests in *N. benthamiana.* As expected, Ha18764 suppressed cell death induced by all the four *H. avenae* inducers (Figure 7). This indicated the cooperation of Ha18764 with other *H. avenae* effectors in regulating plant defenses.

### Ha18764 Suppresses Plant PTI Responses

**FIGURE 8:**
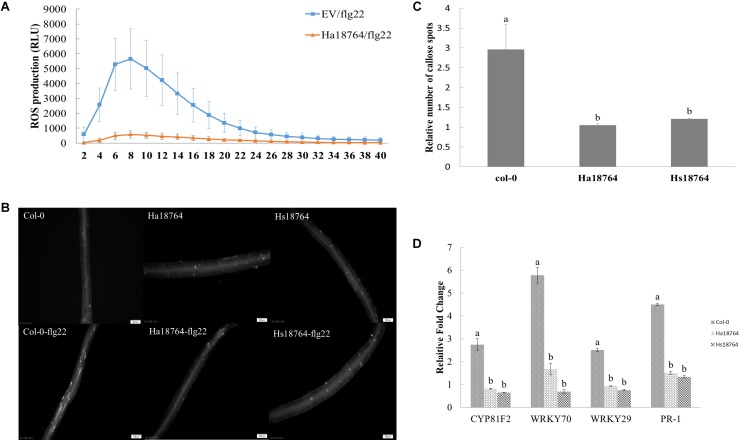
Ha18764 suppresses PTI triggered by flg22. **(A)** Ha18764 suppressed flg22-mediated ROS production in *Nicotiana benthamiana*. The *Agrobacterium tumefaciens* strain GV3101 carrying *Ha18764*, or the empty vector (EV) construct, was infiltrated into *N. benthamiana* leaves. A total of 18–24 infiltrated leaf disks were collected 48 h post-agroinfiltration and assayed for ROS production. **(B)** Ha18764 suppressed callose deposition. *Ha18764* or *Hs18764* transgenic and wild-type (WT, Col-0) *Arabidopsis* plants were treated with flg22; 24 h later, callose (white dots) in the roots was visualized by staining. Images were photographed in the field of approximately 2 mm^2^ that captures the root area containing the root elongation zone. **(C)** Quantified relative numbers of callose. **(D)** Expression of plant defense genes. The mRNA expression levels of *WRKY70*, *WRKY29*, *PR-1*, and *CYP81F2* were measured by qPCR in *Ha18764* or *Hs18764* transgenic and WT plants treated with flg22. Shown are means of three independent biological experiments, each consisting of three technical replicates.

To test whether or not Ha18764 is capable of suppressing a plant’s PTI responses, we measured the ROS production, deposition of cell wall callose and expression levels of defense-related genes after inducing PTI responses by flg22. When challenged with flg22, ROS strongly decreased in the infiltrated *N. benthamiana* leaves expressing *Ha18764* when compared with the empty vector control, which had an obvious induction of ROS production (Figure 8A). Similarly, callose deposition was reduced considerably in the roots of transgenic *Arabidopsis* plants expressing *Ha18764* or *Hs18764* compared with wild-type plants after their treatment with flg22 (Figure 8B,C).

To confirm the PTI-suppression ability of Ha18764, mRNA expression levels of four defense-related genes (*CYP81F2, WRKY70*, *WRKY29*, *PR-1*) were quantified by qPCR in transgenic and wild-type *A. thaliana* plants treated with flg22. As expected, flg22 strongly augmented the expression of these four defense marker genes in wild-type plants, which were 2.5- to 5.8-fold higher than that of the untreated control, whereas the induction levels of these defense genes were significantly repressed in transgenic *Arabidopsis* lines expressing *Ha18764* or *Hs18764* (Figure 8D). This provided direct evidence that Ha18764 can also function as a suppressor of the PTI response in plants.

## Discussion

Some plant-parasitic nematode effectors are present in large multi-gene families, and the G16B09 family is considered among the largest of effector families (Cotton et al., 2014). To date, 11 members from *H. glycines* (Gao et al., 2003; Noon et al., 2015) and 39 members from *G. pallida* (Thorpe et al., 2014) have been reported. Yet, no conserved domains, motifs, or features are predicted from this family, and relatively little is known about their functional roles in nematode–plant interactions. In this study, we demonstrated that several identified members of a G16B09-like family from *H. avenae* suppressed BT-PCD. To our knowledge, the most intensively studied effector family regulating plant defenses is SPRYSEC (secreted proteins containing a SPRY domain), considered to be one of the largest effector families in *G. pallida* (Thorpe et al., 2014). However, SPRYSEC effectors have been implicated in both the suppression and activation of plant immunity (Diaz-Granados et al., 2016). Effector families have also been reported from other plant pathogens; for example, effector sequences are more likely to be found in repeat-rich, gene sparse regions of the genome in *Phytophthora infestans* (Haas et al., 2009). The expanded members in such gene families may reflect the outcome of selection pressure to avoid detection or to maintain key functions within a host.

In *H. avenae*’s G16B09 family, Ha18764 that is most alike to the *H. glycines* G16B09 effector, was selected for further detailed studies. Using the *M. oryzae* secretion system, we found that the SP of Ha18764 was active in guiding the protein product into the cells of the barley leaves. As reported for *H. glycines* and *G. pallida*, the *in situ* hybridization of *Ha18764* revealed dorsal gland localization, additional evidence in support of the secretory ability of *Ha18764*. Usually, dorsal gland cells are more active in the sedentary parasitic stages, when they secrete effectors involved in feeding site formation and maintenance, while subventral gland cells primarily function in secretion during migratory stages, producing proteins required for root invasion and nematode movement within the host plant (Mitchum et al., 2013). Recently, G16B09 family members were found expressed exclusively within the dorsal gland cells in the parasitic stages of *H. glycines* and *G. pallida* (Gao et al., 2003; Thorpe et al., 2014; Noon et al., 2015), thus indicating this gene family’s expression is restricted to the feeding stages. Our results agree with previous reporting on the G16B09 family. Firstly, the hybridization signal of *Ha18764* was detectable only when the J2s were pretreated with host leachates. Secondly, developmental expression pattern analysis showed greater expression of *Ha18764* during the parasitic stages that peak in the J4 stage. So, it is reasonable to presume that Ha18764 functions mainly in the nematode’s parasitic stages.

Due to the narrow host-plant range of *H. avenae* and wheat’s complicated genetic manipulation, both the *Arabidopsis–H. schachtii* infection system and the wheat BSMV-HIGS system were used to investigate Ha18764 functioning during parasitism. Importantly, as a reference, *H. schachtii* infection of the *Arabidopsis* model was employed to verify the role of *H. glycines* effectors in parasitism (Pogorelko et al., 2016; Barnes et al., 2018). The BSMV-HIGS system has been successfully utilized to silence genes in *H. avenae*, by delivering dsRNA from wheat to the nematodes (Chen et al., 2015). In our present study, both *Ha18764*- and *Hs18764-*transgenic *Arabidopsis* lines were more susceptible to *H. schachtii* infection than wild-type plants. Meanwhile, silencing of *Ha18764 in vivo* using the BSMV-HIGS system significantly impeded nematode infection of wheat. These results further confirmed that Ha18764 has a role to play in parasitism.

Cyst nematodes are considered as biotrophic pathogens, because they feed from the syncytia until their reproduction is complete. Therefore, *H. avenae* needs to suppress plant defenses during the entire parasitic process. For survival, *H. avenae* must possess the ability to suppress the host defenses including PTI and ETI. Our results showed that Ha18764 could suppress both PTI and ETI. The PTI assay showed that Ha18764 could indeed suppress the deposition of callose and the production of ROS. Since Ha18764 was able to suppress programmed cell death induced by the R-protein/cognate effector pairs (i.e., R3a/Avr3a and RBP-1/Gpa2), this suggested Ha18764 suppresses plant ETI responses. Several nematode effectors are reported to be capable of suppressing both ETI and PTI. For example, The CEP12 peptide from *Globodera rostochiensis* suppresses both resistance-gene-mediated cell death and flg22-mediated ROS production (Chen et al., 2013; Chronis et al., 2013). The *M. incognita* putative secretory protein MiMsp40 suppresses cell death triggered by the ETI elicitors R3a/Avr3a, and overexpression of *MiMsp40* in plants suppresses the deposition of callose and the expression of PTI marker genes (Niu et al., 2016). Moreover, Ha18764 also suppressed two MAPK kinases genes (MKK1 and NPK1) triggering cell death in *N. benthamiana*. Plant MAPK cascade pathways play remarkably important roles in plant defense signaling (Dodds and Rathjen, 2010; Tena et al., 2011), and they are considered crucial for PTI and ETI responses (Meng and Zhang, 2013). Our results indicate Ha18764 specifically targets a point downstream of MKK1 and NPK1 in the signaling pathway. Furthermore, Ha18764 could suppress the expression of a suite of key plant defense-related genes that are mainly involved in the SA signaling pathway. The *Arabidopsis CYP81F2* gene encodes a P450 monooxygenase that is essential for antimicrobial defense (Bednarek et al., 2009). WRKY70 functions as an activator of salicylic acid (SA)-induced genes and as a repressor of jasmonic acid (JA)-responsive genes (Li et al., 2004). The flg22-triggered transcription of *WRKY29* was recently shown to depend on SA signaling (Yi et al., 2014). *PR-1* is commonly used as molecular marker for SA-dependent systemic acquired resistance signaling (Bowling et al., 1994). Accordingly, we hypothesize Ha18764 interferes with the SA signaling pathway to suppress host immune responses. SA-dependent signaling is considered to be crucial for resistance against biotrophic pathogens (Delaney et al., 1994). For example, the nematode *H. schachtii* reportedly elicits SA-dependent plant resistance in both roots and leaves of infected *Arabidopsis* (Hamamouch et al., 2011). Because our results are consistent with these previous reports, we propose that Ha18764 interferes with SA-dependent plant resistance to promote nematode parasitism.

Consistent with our recent work (Chen C. et al., 2018), evidence for interaction between Ha18764 and *H. avenae* putative effectors for cell death induction was found. Ha18764 also suppressed cell death triggered by four *H. avenae* cell-death-inducing effectors. The similar interaction between effectors was also found in SPRYSECs effectors of *G. pallida* and RxLR effectors of *P. sojae* (Wang et al., 2011; Mei et al., 2015). Furthermore, in both *P. sojae* and *P. parasitica*, effector XEG1 is bound by host-secreted GmGIP1 which blocked its contribution to virulence; however, these pathogens secrete a paralogous XLP1 that binds to GmGIP1 more tightly than does PsXEG1, thus freeing XEG1 to support the infection process (Ma et al., 2017). It is possible the same mechanism exists in *H. avenae*. Diversity in the effector family may have arisen from selection pressure to escape recognition by hosts. Through its interacting effectors, *H. avenae* effectively avoided inducing host defenses against its biotrophic parasitism.

In summary, we have identified a pioneer effector family from the nematode *H. avenae* and verified that the majority of the G16B09-like family members could suppress BT-PCD. One novel family member in particular, Ha18764, suppresses various defense responses as a secreted effector during interaction with plants. Our study suggests Ha18764 may indeed benefit parasitism by suppressing host-plant immunity encompassing diverse PTI and ETI pathways. Further study of these family members and their interactions with receptors in host cells may reveal the molecular mechanism underlying plant defense suppression.

## Author Contributions

HJ and QL conceived the idea for this study, acquired its funding, and designed the experiments. SY, YD, and YC performed the experiments. DY carried out the bioinformatics analyses. JY provided the pRP27-mCherryNLS system. QL, SY, and HJ wrote and revised the manuscript. All authors read and approved the final version of the manuscript for publication.

## Conflict of Interest Statement

The authors declare that the research was conducted in the absence of any commercial or financial relationships that could be construed as a potential conflict of interest.

## Footnotes

1 http://www.ncbi.nlm.nih.gov/Structure/cdd/wrpsb.cgi

2 http://www.cbs.dtu.dk/services/SignalP

3 http://www.cbs.dtu.dk/services/TMHMM/

4 https://www.genscript.com/psort.html

5 http://imagej.nih.gov/ij/

## Supplementary Material

FIGURE S1 Phylogenetic tree of G16B09 family members from different nematodes. For *G. pallida*, the genomic gene numbers are listed. For *H. glycines* and *H. avenae*, the GenBank accession numbers are listed after the gene names. Hg, *H. glycines*; Ha, *H. avenae*; Gp, *G. pallida*.

FIGURE S2 Suppression of BT-PCD by *Heterodera avenae* G16B09-like effector family in *Nicotiana benthamiana*. The *N. benthamiana* leaves were infiltrated with a buffer, or *A. tumefaciens* cells carrying the candidate gene, or the negative control eGFP gene, either alone or 24 h prior to infiltration with *A. tumefaciens* cells carrying *Bax*. Photographs of infiltrated leaves were taken ca. 4 days after the last infiltration. Numbers in parentheses indicate the proportion of infiltrated sites showing cell-death-suppressing symptoms. Shown below is the verified protein expression of *H. avenae* proteins, eGFP, and Bax, by Western blotting.

FIGURE S3 The original Western blotting images embedded into Figure 6. The expression of GFP or Ha18764 were verified using anti-flag. All bands are labeled with a black line.

FIGURE S4 The original Western blotting images embedded into Figure 6. The expression of MKK1, NPK1^Nt^ or NIP were verified using anti-HA. All bands are labeled with a black line.

FIGURE S5 The original Western blotting images embedded into Figure 7. The expression of GFP or Ha18764 were verified using anti-flag. All bands are labeled with a black line.

FIGURE S6 The original Western blotting images embedded into Figure 7. The expression of Ha16511, Ha19390, Ha12969 or Ha16978 were verified using anti-HA. All bands are declared with a black line.

FIGURE S7 Sequence analysis of *Heterodera avenae* G16B09-like effector family. **(A)** Alignment of *H. avenae* G16B09-like effector family. **(B)** Phylogenetic tree of G16B09-like family members from *H. avenae*.

TABLE S1 List of all the primer sequences used in this study.
