# Supplementary figures and images for: A Novel G16B09-Like Effector From Heterodera avenae Suppresses Plant Defenses and Promotes Parasitism
Source: Front Plant Sci. 2019 Feb 8;10:66. doi: 10.3389/fpls.2019.00066 (PMC6376208; doi:10.3389/fpls.2019.00066)

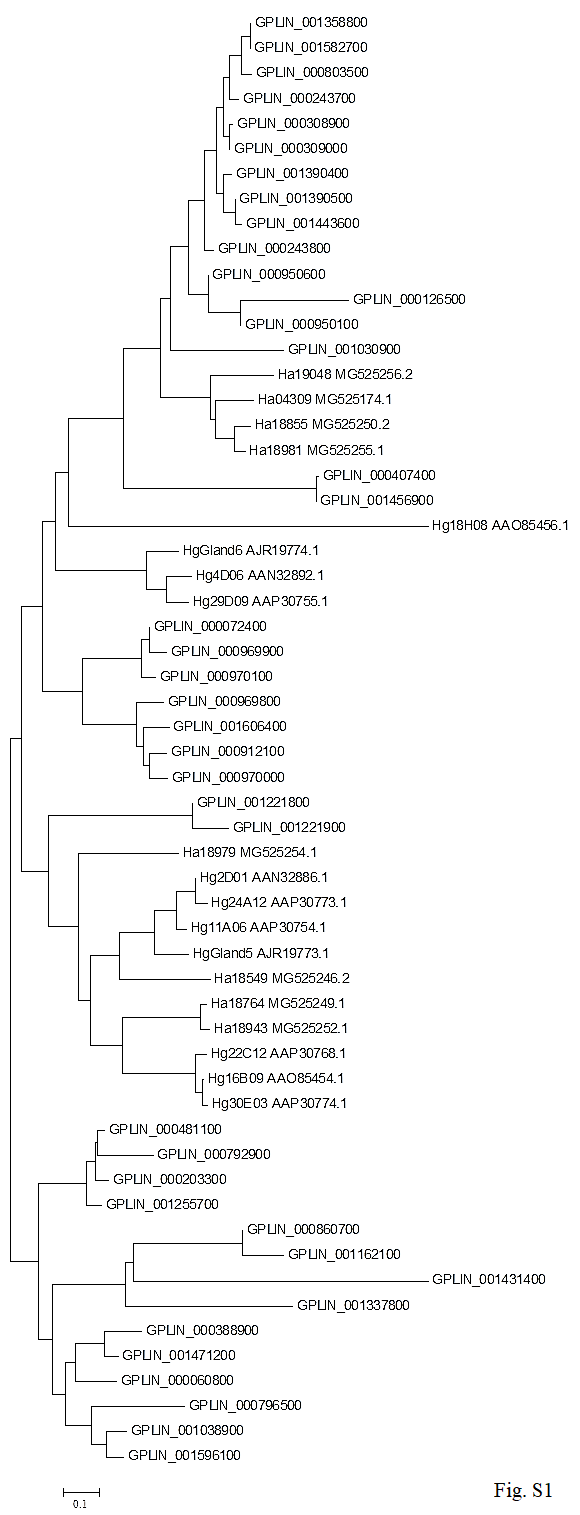

Supplement: FIGURE S1 — Phylogenetic tree of G16B09 family members from different nematodes. For G. pallida, the genomic gene numbers are listed. For H. glycines and H. avenae, the GenBank accession numbers are listed after the gene names. Hg, H. glycines; Ha, H. avenae; Gp, G. pallida. [file Image_1.TIF]

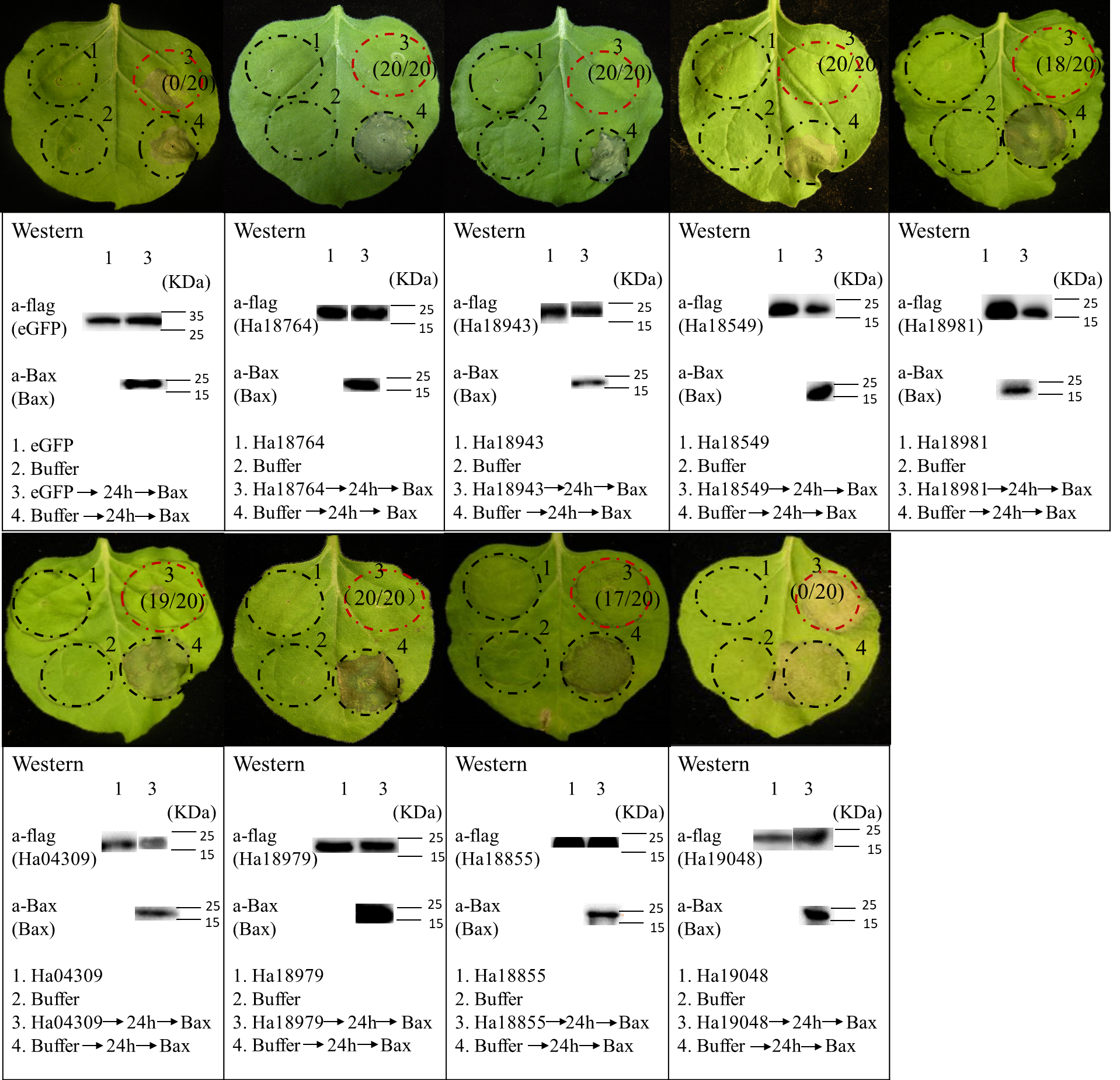

Supplement: FIGURE S2 — Suppression of BT-PCD by Heterodera avenae G16B09-like effector family in Nicotiana benthamiana. The N. benthamiana leaves were infiltrated with a buffer, or A. tumefaciens cells carrying the candidate gene, or the negative control eGFP gene, either alone or 24 h prior to infiltration with A. tumefaciens cells carrying Bax. Photographs of infiltrated leaves were taken ca. 4 days after the last infiltration. Numbers in parentheses indicate the proportion of infiltrated sites showing cell-death-suppressing symptoms. Shown below is the verified protein expression of H. avenae proteins, eGFP, and Bax, by Western blotting. [file Image_2.tif]

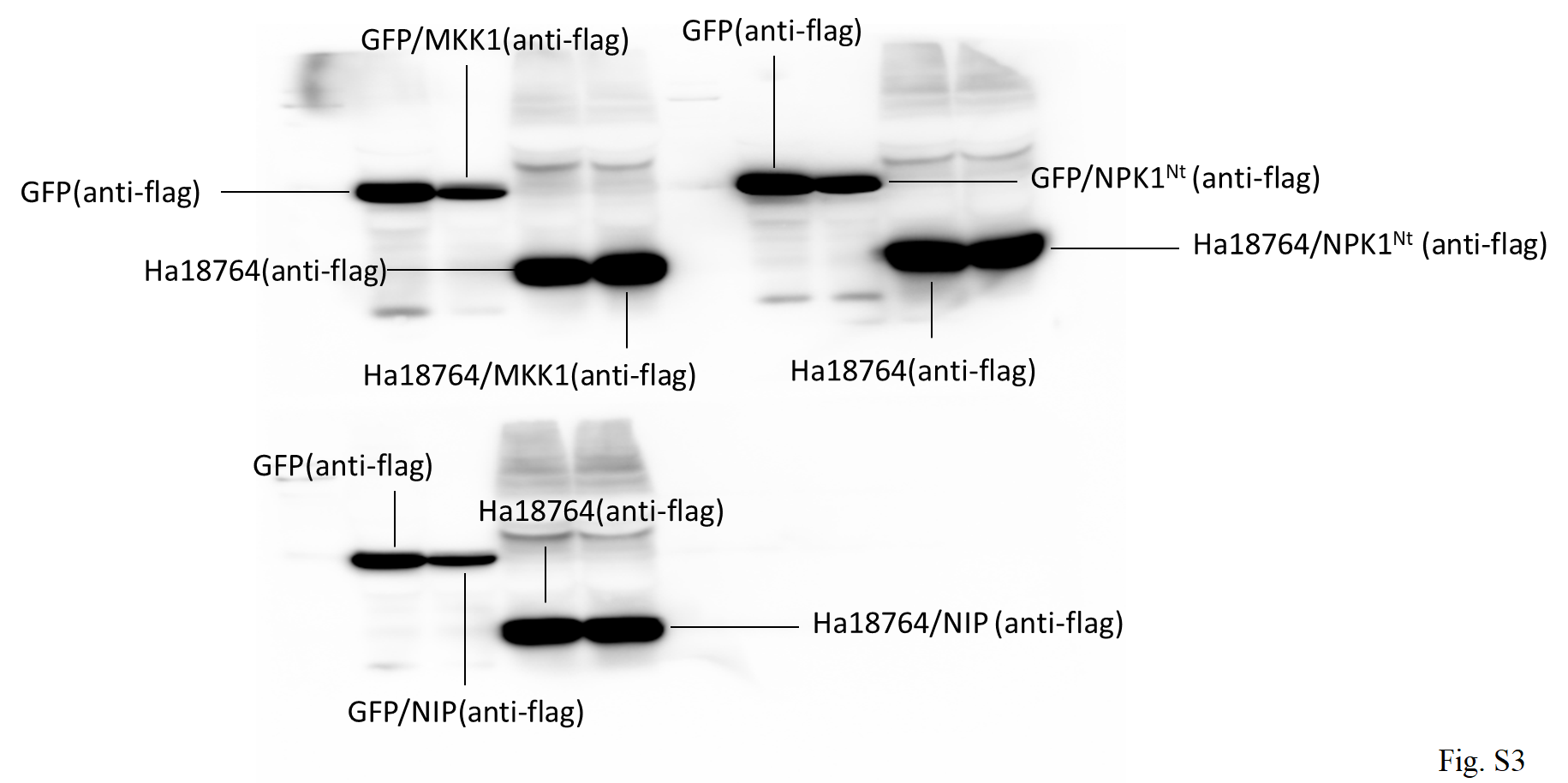

Supplement: FIGURE S3 — The original Western blotting images embedded into Figure 6. The expression of GFP or Ha18764 were verified using anti-flag. All bands are labeled with a black line. [file Image_3.TIF]

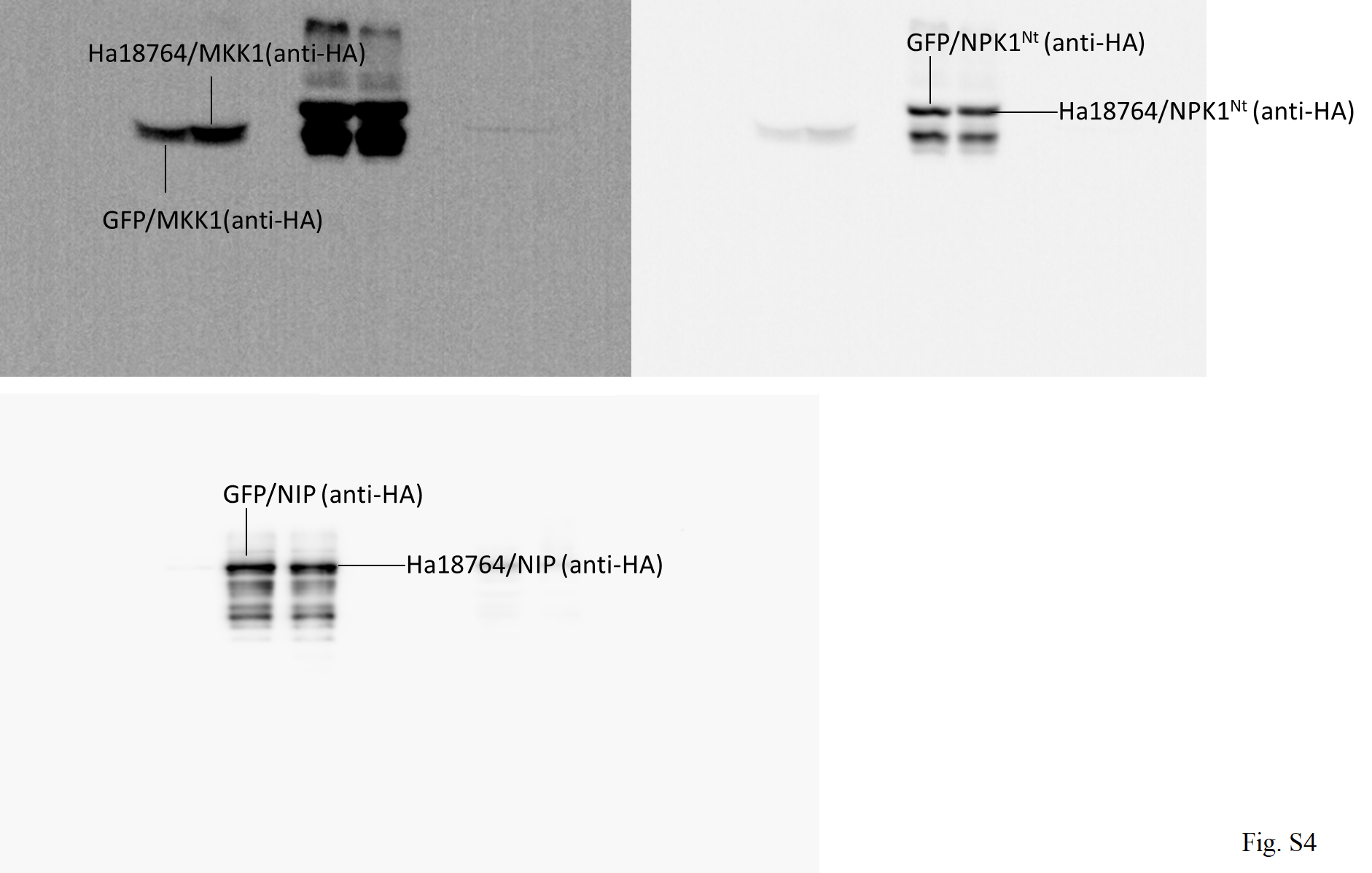

Supplement: FIGURE S4 — The original Western blotting images embedded into Figure 6. The expression of MKK1, NPK1Nt or NIP were verified using anti-HA. All bands are labeled with a black line. [file Image_4.TIF]

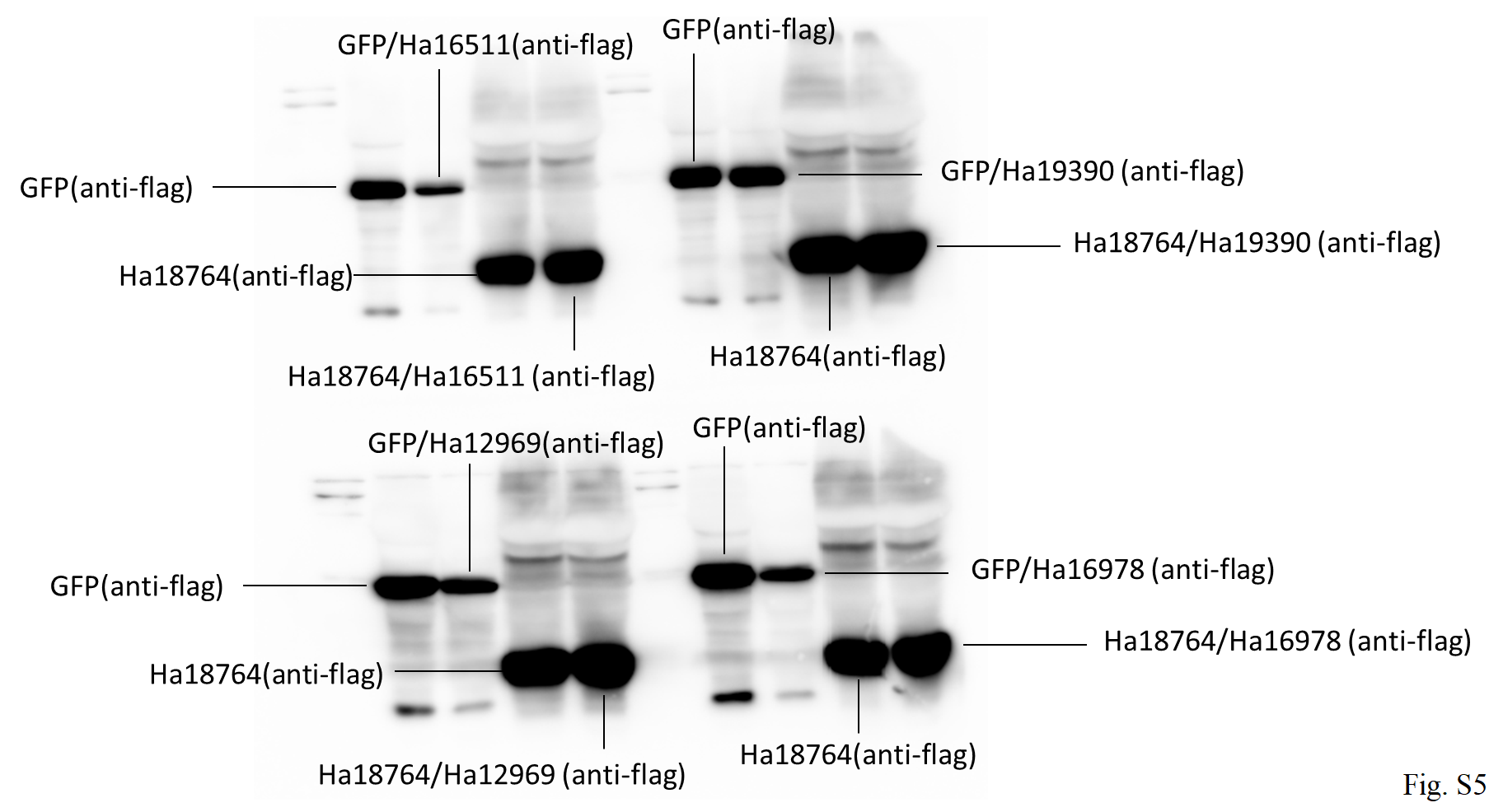

Supplement: FIGURE S5 — The original Western blotting images embedded into Figure 7. The expression of GFP or Ha18764 were verified using anti-flag. All bands are labeled with a black line. [file Image_5.TIF]

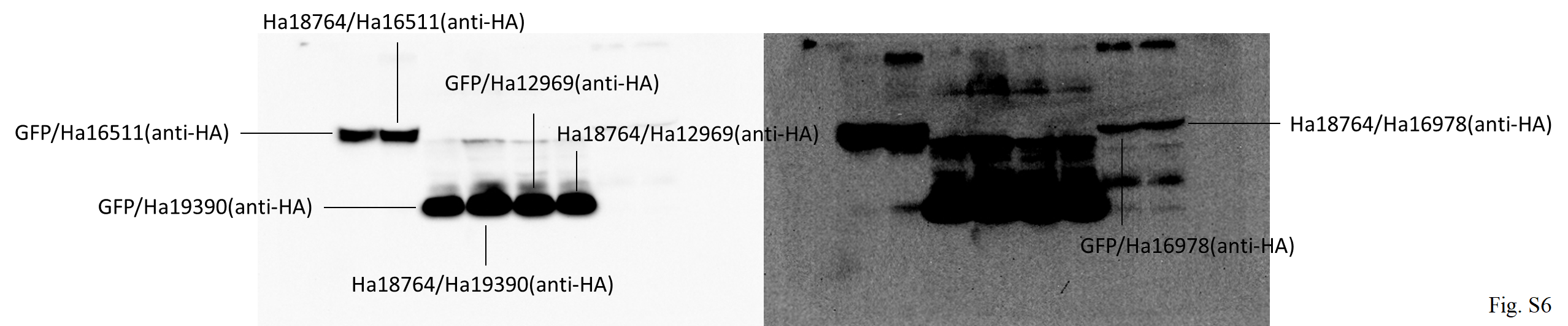

Supplement: FIGURE S6 — The original Western blotting images embedded into Figure 7. The expression of Ha16511, Ha19390, Ha12969 or Ha16978 were verified using anti-HA. All bands are declared with a black line. [file Image_6.TIF]

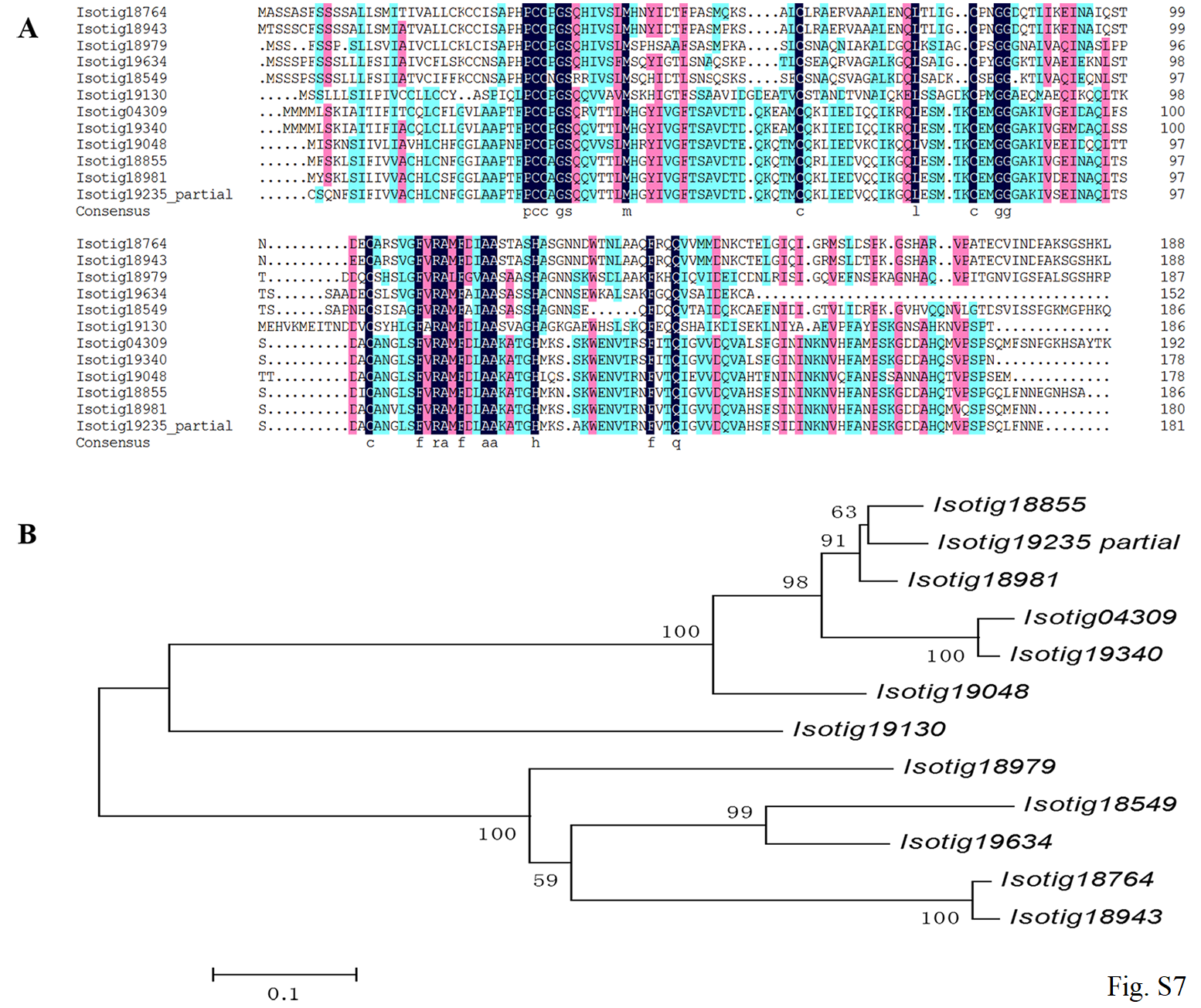

Supplement: FIGURE S7 — Sequence analysis of Heterodera avenae G16B09-like effector family. (A) Alignment of H. avenae G16B09-like effector family. (B) Phylogenetic tree of G16B09-like family members from H. avenae. [file Image_7.TIF]
