# Supplementary material for: A Novel G16B09-Like Effector From Heterodera avenae Suppresses Plant Defenses and Promotes Parasitism
Source: Front Plant Sci. 2019 Feb 8;10:66. doi: 10.3389/fpls.2019.00066 (PMC6376208; doi:10.3389/fpls.2019.00066)
Supplement: TABLE S1 — List of all the primer sequences used in this study. [file Table_1.DOCX]

Table S1 Primer sequences used in this study

| **Primer name** | **Sequence (5'-3')** | **application** |
| --- | --- | --- |
| *Ha18764*cds-F | ATGGCTTCTTCAGCATCCTTTT | gene amplification |
| *Ha18764*cds-R | TCAGAGTTTGTGTGATCCGCTT |  |
| *Hg18764*cds-F | ATGGCTTCATCTTTCTGCT |  |
| *Hg18764*cds-R | TTAAATGCGCTTGTGAGAC |  |
| *Ha18764*qPCR-F | ATGGCTTCTTCAGCATCCTTTTCC | qPCR |
| *Ha18764*qPCR-R | GCGAGACAATGTGTTGGCTACC |  |
| *HaGAPDH-1*-F | AGCGGCACAGAACATCATCC |  |
| *HaGAPDH-1*-R | GGTCCTCCGTGTAGCCCAAA |  |
| *in-situ-Ha18764*-F | ATGGCTTCTTCAGCATCCTTTTC | *in situ* hybridization |
| *in-situ-Ha18764*-R | GAGTTTGTGTGATCCGCTTTTG |  |
| *Ha18764*dsp-F | TATCCCGGGATGGCACCCCACCCGTGC | subcellular localization |
| *Ha18764*dsp-R | GTCGTCGACGAGTTTGTGTGATCCGCT |  |
| *Ha18764*VaF | GTCGACGGTATCGATAAGCTTATGGCTTCTTCAGCATCCTTTT | SP validation |
| *Ha18764*VaR | CGCTCTAGAACTAGTGGATCCGAGTTTGTGTGATCCGCTT |  |
| *Ha18764*RNAi-F | AAGGAAGTTTAATCAATTGGTTTTCCAAGGCCGC | VIGS |
| *Ha18764*RNAi-R | AACCACCACCACCGTTCCTCTGCTCTTCTTTCCATG |  |
| *Q-HIG-18764-F* | CGCAGTGTCGGCTTTGTC |  |
| *Q-HIG-18764-R* | GTCGGAACTGAGCAGCCAAG |  |
| 18764-F | ACTAGTGGATCCCCCGGGATGGCACCCCACCCGTG | cell death suppression |
| 18764-R | TTCATCGGCGGTCGACTCAGAGTTTGTGTGATC |  |
| 18943-F | *ACTAGTGGATCCCCCGGG*ATGGCACCCCACCCGTG |  |
| 18943-R | *TTCATCGGCGGTCGAC*TCACAGTTTGTGTGATC |  |
| 18979-F | *TAGTGGATCCCCCGGG*ATGGCACCCCATCCG |  |
| 18979-R | *TTCATCGGCGGTCGAC*TTATAAGGGCCGATG |  |
| 04309-F | *ACTAGTGGATCCCCCGGG*ATGGCTCCAACTTTTCC |  |
| 04309-R | *TTCATCGGCGGTCGAC*TTATTTTGTGTAGGCGC |  |
| 18549-F | *ACTAGTGGATCCCCCGGG*ATGGCACCCCATCCGTG |  |
| 18549-R | *TTCATCGGCGGTCGAC*TCATTGTTTGTGCGGGC |  |
| 04309-F | *ACTAGTGGATCCCCCGGG*ATGGCTCCAACTTTTCC |  |
| 04309-R | *TTCATCGGCGGTCGAC*TTATTTTGTGTAGGCGC |  |
| 18855-F | *ACTAGTGGATCCCCCGGG*ATGGCTCCAACTTTCCCG |  |
| 18855-R | *TTCATCGGCGGTCGAC*CTATGCGCTATGGTTGC |  |
| 19048-F | *ACTAGTGGATCCCCCGGG*ATGGCTCCAAATTTTCC |  |
| 19048-R | *TTCATCGGCGGTCGAC*CTACATTTCACTCGGAC |  |
| 18981-F | *ACTAGTGGATCCCCCGGG*ATGGCACCAACTTTTCC |  |
| 18981-R | *TTCATCGGCGGTCGAC*TTAGTTGTTGAACATTT |  |
| psojNIP-F | CACCAGCTAGCATCGATATGAACCTCCGCCCTG | cell death inducer |
| psojNIP-R | TATGGGTACGCGGCCGCAGCGTAGTAGGCGTTG |  |
| 16511-F | CTAGCACGCGTATCGATATGTTTTTCATCGGTTC |  |
| 16511-R | ATGGTGATGGTGATGATGGCCATTGTTCGTAATCCGTC |  |
| 19390-F | CTAGCACGCGTATCGATATGTCTTGGCCTGATCTTGTCA |  |
| 19390-R | TCAATGGTGATGGTGATGATGACCGTAATTAATGCTTCGG |  |
| 12969-F | CTAGCACGCGTATCGATATGAGTGGGTGGGATGC |  |
| 12969-R | ATGGTGATGGTGATGATGGCCGTAACCGGCGTCCTCGAG |  |
| 16978-F | CTAGCACGCGTATCGATATGACACCAACTCCAGTGATGC |  |
| 16978-R | TTAATGGTGATGGTGATGATGACCAAAAAGTTGAAATGAC |  |
| 1300-Ha18764-F | CAAATCGACTCTAGAAAGCTTATATGGCACCCCACCCGTGCT | ectopic expression in *Arabidopsis* |
| 1300-Ha18764-R | GTCTTTGTAGTCCATGGTACCGAGTTTGTGTGATCCGCTTTTGG |  |
| 1300-Hs18764-F | CAAATCGACTCTAGAAAGCTTATATGGCACCCCATACGTGCT |  |
| 1300-Hs18764-R | GTCTTTGTAGTCCATGGTACCAATGCCGCTGTGAGACCCG |  |
| 1132-Ha18764-F | CGCTCTAGAACTAGTGGATCCATGGCACCCCACCCGTGCT | ROS assay |
| 1132-Ha18764-R | GGGCCCCCCCTCGAGGTCGACGAGTTTGTGTGATCCGCTTTTGG |  |
| WRKY70-F | CATACATAGGAAACCACACG | Defense-related gene expression |
| WRKY70-R | CTCCAAACACCATGAGATCC |  |
| WRKY29-F | ATCCAACGGATCAAGAGCTG |  |
| WRKY29-R | GCGTCCGACAACAGATTCTC |  |
| PR1-F | TTCACAACCAGGCACGAGGAG |  |
| PR1-R | GCCAGACAAGTCACCGCTACC |  |
| CYP81F2-F | GTGAAAGCACTAGGCGAAGC |  |
| CYP81F2-R | ATCCGTTCCAGCTAGCATCA |  |
| actin_F | AGTGGTCGTACAACCGGTATTGT |  |
| actin_R | GAGGATAGCATGTGGAACTGAGAA |  |
